# P-495. Anal Cancer Screening and Referral Practices in an Academic Medical Center Affiliated HIV Clinic in Columbus, Ohio

**DOI:** 10.1093/ofid/ofae631.694

**Published:** 2025-01-29

**Authors:** Syeda F Hassan, Ashley Lipps, Yesha Patel, Mohammad Mahdee Sobhanie, Carlos Malvestutto, Susan L Koletar

**Affiliations:** Ohio State University, Hilliard, Ohio; The Ohio State University Wexner Medical Center, Columbus, Ohio; The Ohio State University Wexner Medical Center, Columbus, Ohio; The Ohio State University, Columbus, Ohio; The Ohio State University Wexner Medical Center, Columbus, Ohio; Ohio State University, Hilliard, Ohio

## Abstract

**Background:**

Rates of anal cancer (AC) in people living with HIV (PLWH) are higher than the general population. Screening for AC can be performed using cytology +/- high-risk human papillomavirus (hrHPV) testing. Until recently, there were no evidence-based consensus guidelines available regarding who should be screened for AC or management of abnormal cytology results. The International Anal Neoplasia Society (IANS) guidelines recommend AC screening in PLWH starting at age 35 for men and transgender women who have sex with men (MSM) and starting at age 45 for other PLWH. Guidelines recommend referrals for high resolution anoscopy (HRA) in those with cytology results of atypical squamous cells of unknown significance (ASCUS)/hrHPV+ or worse. The objective of this study is to evaluate existing AC screening and HRA referral practices in the Ohio State University Wexner Medical Center (OSUWMC) Infectious Diseases Clinic (IDC) to identify gaps in care based on the new IANS guidelines.

**Methods:**

This was a single-center, retrospective study evaluating PLWH age >18 years old who underwent AC screening at the OSUWMC IDC between October 1, 2020 and September 30, 2023. Demographic and clinical outcomes were obtained.

**Results:**

During the study period, 257 PLWH had anal cytology performed. Demographic information is shown in Table 1. Of 257 patients screened, median age was 49. 250 patients (97.3%) were men, including 241 (93.7%) MSM. 241 patients (93.8%) had HIV viral load < 40 copies/mL and 251 (97.7%) had CD4 counts >200 cells/mm3. Clinical outcomes, including cytology results, referrals to colorectal surgery (CRS) and results of HRA are shown in Table 2. Of patients with ASCUS/hrHPV+ or worse on cytology, 60/67 (90%) were referred to CRS and 31/67 (46%) underwent HRA.

**Figure:**
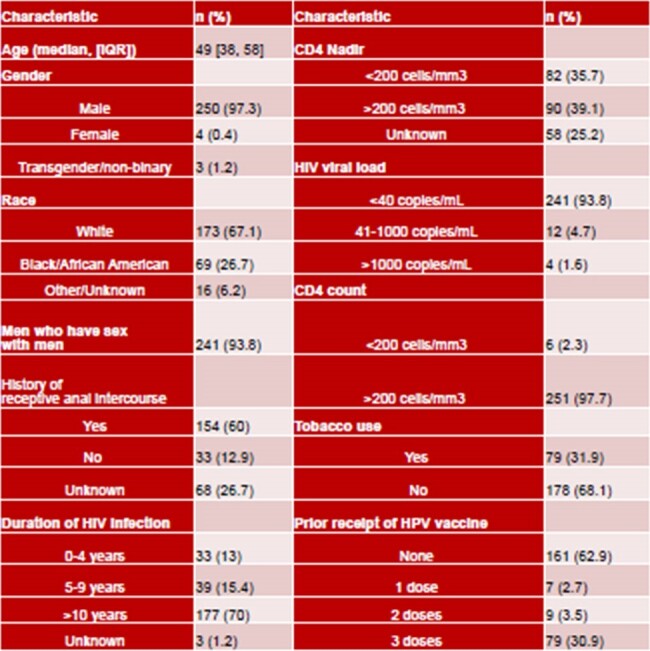

**Figure:**
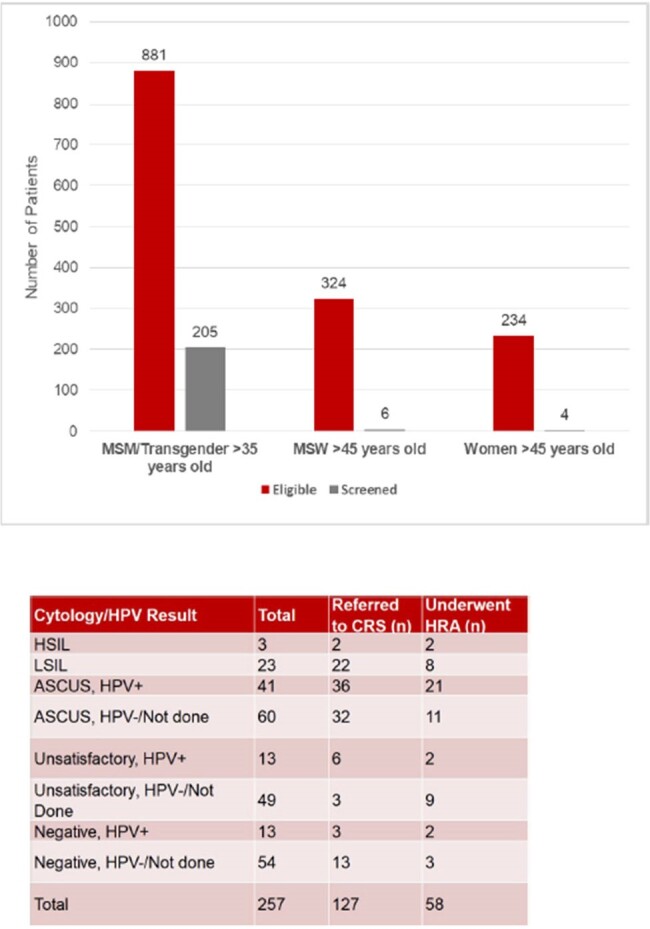

**Figure:**
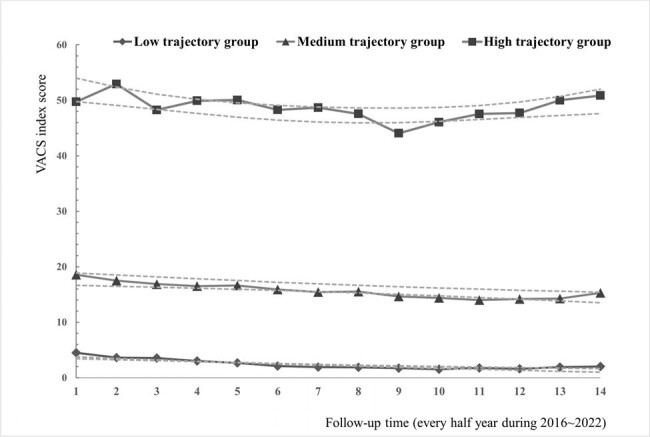

**Conclusion:**

Anal cancer screening was completed predominantly in MSM living with HIV. Further efforts are needed to promote anal cancer screening among other at-risk groups, such as women and MSW living with HIV. CRS referral practices based on cytology results were highly variable. Though most patients with ASCUS/hrHPV+ or worse were referred to CRS for HRA (consistent with new guidelines), less than half completed an HRA. Additional investigations are needed to determine individual and system level barriers to HRA in this patient population.

**Disclosures:**

**Carlos Malvestutto, MD MPH**, Gilead Sciences: Advisor/Consultant|Viiv Healhcare: Advisor/Consultant

